# Microflow Nanoprecipitation of Positively Charged Gastroresistant Polymer Nanoparticles of Eudragit^®^ RS100: A Study of Fluid Dynamics and Chemical Parameters

**DOI:** 10.3390/ma13132925

**Published:** 2020-06-30

**Authors:** Cristina Yus, Manuel Arruebo, Silvia Irusta, Victor Sebastián

**Affiliations:** 1 Department of Chemical Engineering and Environmental Technology, Aragon Institute of Nanoscience (INA), Instituto de Ciencia de Materiales de Aragon-ICMA, University of Zaragoza, 50018 Zaragoza, Spain; cyargon@gmail.com (C.Y.); sirusta@unizar.es (S.I.); 2 Networking Research Center on Bioengineering Biomaterials and Nanomedicine (CIBER-BBN), 28029 Madrid, Spain

**Keywords:** nanoparticle, nanoprecipitation, micromixing, antibiotic

## Abstract

The objective of the present work was to produce gastroresistant Eudragit^®^ RS100 nanoparticles by a reproducible synthesis approach that ensured mono-disperse nanoparticles under the size of 100 nm. Batch and micromixing nanoprecipitation approaches were selected to produce the demanded nanoparticles, identifying the critical parameters affecting the synthesis process. To shed some light on the formulation of the targeted nanoparticles, the effects of particle size and homogeneity of fluid dynamics, and physicochemical parameters such as polymer concentration, type of solvent, ratio of solvent to antisolvent, and total flow rate were studied. The physicochemical characteristics of resulting nanoparticles were studied applying dynamic light scattering (DLS) particle size analysis and electron microscopy imaging. Nanoparticles produced using a micromixer demonstrated a narrower and more homogenous distribution than the ones obtained under similar conditions in conventional batch reactors. Besides, fluid dynamics ensured that the best mixing conditions were achieved at the highest flow rate. It was concluded that nucleation and growth events must also be considered to avoid uncontrolled nanoparticle growth and evolution at the collection vial. Further, rifampicin-encapsulated nanoparticles were prepared using both approaches, demonstrating that the micromixing-assisted approach provided an excellent control of the particle size and polydispersity index. Not only the micromixing-assisted nanoprecipitation promoted a remarkable control in the nanoparticle formulation, but also it enhanced drug encapsulation efficiency and loading, as well as productivity. To the best of our knowledge, this was the very first time that drug-loaded Eudragit^®^ RS100 nanoparticles (NPs) were produced in a continuous fashion under 100 nm (16.5 ± 4.3 nm) using microreactor technology. Furthermore, we performed a detailed analysis of the influence of various fluid dynamics and physicochemical parameters on the size and uniformity of the resulting nanoparticles. According to these findings, the proposed methodology can be a useful approach to synthesize a myriad of nanoparticles of alternative polymers.

## 1. Introduction

In recent years, the synthesis of polymeric nanoparticles (NPs) has gained wide recognition in biomedical applications [1,2,3], playing a pivotal role in a wide variety of pharmaceutical applications such as diagnostics and drug delivery [4]. These NPs are designed to transport and deliver active components such as drugs [5], active metals [6], contrast agents [7] and biomolecules [8] within biological systems. One of the advantages of polymeric NPs is that polymer properties can be attuned on demand, selecting the type of functionalization, biodegradability rate, hydrophilicity, biocompatibility, and the type of response to specific physiological or external stimuli. When polymer nanoparticles are used as drug delivery systems it is of paramount importance for the control of synthesis conditions to attune the system specifications with the targeted pathology. In this sense, size, shape, chemical composition, and surface charge are some of the key features that drug delivery systems should consider to achieve a proper biodistribution, as well as high circulation time, blood stability, and controlled drug release [9,10,11]. On the other hand, the design of polymeric NPs for a therapeutic use should also consider the payload required, the targeted site of action, and the route of administration. In this sense, the polymer properties can substantially differ if an intravenous or an oral administration were considered.

In the design of polymer drug delivery nanosystems, it is well accepted that sub 100 nm particles have longer circulation half-lives than their larger counterparts because their rate of clearance is decreased [12]. Furthermore, they avoid a fast clearance by the immune system because they are not recognized by macrophages as strange bodies [13]. To achieve a long circulation half-life, the surface charge of the nanosystem must also be taken into account. By controlling the surface charge, it is feasible to suppress protein binding and the consequent rapid clearance by the immune system cells [14,15].

Positively charged polymers are considered as mucoadhesive materials that are based on the ionic interaction between the polymer and the mucosal membrane, which is negatively charged [16]. Positively charged Eudragit^®^ polymers have been extensively used in the design of mucoadhesive polymeric carriers [17,18]. Eudragit^®^ polymers are synthetically obtained by the polymerization of acrylic acid, methacrylic acids, and their esters. The ratio of monomers is a key variable to control the physicochemical characteristics of Eudragit^®^ polymers, as well as their drug release behavior. Mucoadhesive pH-independent Eudragit^®^ polymers can be classified according to their permeability and swellability in the release media [19]: Eudragit^®^ RL (highly permeable), Eudragit^®^ RS (low permeable), Eudragit^®^ NE (permeable), and Eudragit^®^ NM (permeable). Eudragit^®^ RS100 is a co-polymer of poly (ethylacrylate, methyl-methacrylate, chlorotrimethylammonioethyl methacrylate) containing around 4.5–6.8% of quaternary ammonium groups that impart a positive charge to this biocompatible polymer [20]. This fact is pertinent to maximizing the cellular uptake since the cellular surface of the target tissues is negatively charged [21]. The suitability of this polymer for the design of mucoadhesive drug delivery vectors [22] as targeted delivery systems to oral [23], sublingual [24], intestinal [25], dermal [26], ophthalmologic [27], or vaginal [28] mucosa has been reported. Eudragit^®^ RS100 has been commonly well-considered for the preparation of controlled-release oral pharmaceutical dosage forms [29] because it is insoluble at physiological pH values. Therefore, this polymer is an excellent candidate to protect the therapeutic cargo from the enzymatic and acidic degradation (pH approx. 1.5–4) that takes place in the stomach [30]. Both gastrointestinal protection and mucoadhesion may lengthen the contact of polymeric NPs with the intestinal epithelium and should be enough to increase the bioavailability of the loaded active pharmaceutical ingredient.

To achieve the desired physicochemical properties in polymeric NPs different synthesis methods are available, for example, salting out [31], nanoprecipitation [32], spray drying [33], or simple or double emulsion [34]. Eudragit^®^ RS100 NPs have been produced by nanoprecipitation [35], spray drying [36], and emulsification [37] techniques, but as far as we know from the literature, the particle size distribution achieved was not feasible to be decreased under the 100-nm barrier. From the aforementioned synthesis approaches, nanoprecipitation methodology (also called solvent displacement method) is quite convenient because it is simple, one-step, rapid, and requires low energy. The nanoprecipitation process generally yields nanoparticles by the interfacial diffusion of a polymer dissolved in a semipolar solvent (e.g., acetone) into a high miscible solvent (antisolvent) where the polymer is not soluble (e.g., water) [38]. When the solvent is displaced by the antisolvent, a burst nucleation of nanoparticles occurs because of the lack of solubility of the polymer in the antisolvent and the increase in the supersaturation [39]. When supersaturation decreases and no additional nucleation events occur, the size of the nuclei gradually increases. Finally, nanoparticles are formed by coalescence/aggregation events until the polymer concentration decreases to reach the solubility concentration [40]. This simple process has two main constraints: (1) The semipolar solvent should be easily evaporated (low boiling point) to avoid the thermal degradation of the payload and should have a low residual toxicity according to the pharmacopeia (class 3 solvents are preferred against class 1 solvents) [41]. (2) The fluid dynamics and solvents’ miscibility in the nanoprecipitation process will govern the mixing process and nucleation/growth rates. Then, the production of polymeric NPs by nanoprecipitation is conditioned by the injection rate of the solvent/antisolvent, the mixing rate, the nature of the solvents and their interactions, and the presence of surfactants that control the particle size and functionality. Normally, the organic solvents considered are ethanol or acetone, which have a low toxic potential (considered as class 3 solvents by the European Medicines Agency).

As aforementioned, one of the principal limitations of the nanoprecipitation methodology is the control of the hydrodynamics, which means that the mixing rate is a very important parameter in the resulting NP size distribution [42]. Nanoprecipitation in batch-type reactors is usually performed either via one-pot by adding the polymer solution into the non-solvent, or by a controlled dropwise addition of one phase into the other [43]. Independent of the selected strategy, batch-type system hydrodynamics suffer from serious limitations, presenting low controllability of physicochemical parameters. Some procedures based on batch-type reactors have dealt with the poor mixing by lengthening the reaction time, using surfactants to slow down the release of reactants and consequently controlling the nucleation/growth kinetics [44]. In contrast, microfluidics offers the possibility of reducing nucleation/growth times by reducing diffusional distances by a few tens of micrometers. The improved diffusion results in a very fast mixing achieved within a few milliseconds down to microseconds [45]. Microfluidic systems are powerful tools to carry out fast kinetic chemical reactions, such as nanocrystallization. These reaction devices enable the manipulation of small quantities of fluids, passing them through microchannels. The sub-millimeter dimensions of fluid channels lead to a relatively large surface area-to-volume ratio that improves heat and mass transport. It is in the control of the grade of mixing and the reproducibility of the synthesis where microfluidics plays an important role, allowing for higher throughput and better process control [46]. Micromixers are a special type of microfluidic system designed to efficiently modify the fluid streamlines by molecular diffusion, convection, and chaotic advection [47]. Thus, the selection of a micromixer configuration and inlet flow rates can yield different physicochemical properties in the resulting NPs [48]. In addition, micromixers operate in continuous flow while maintaining over time the same product specifications, which is an advantage to scale-up the production and translate it from the lab to the market [49].

In the present work we propose micromixer-assisted nanoprecipitation to study the effect of fluid dynamics and physicochemical parameters on the synthesis of polymer nanoparticles of Eudragit^®^ RS100. The main objective was to use a continuous flow synthesis approach for formulating monodispersed NPs, with controlled particle size under 100 nm to achieve long circulation half-life and decrease the rate of clearance. The effects of operational fluidic conditions, solvents, feed concentration of polymer, and solvent/antisolvent ratio on the size characteristics of resulting NPs were examined. Rifampicin, an antibiotic, was considered as a proof of concept to be encapsulated in the continuous flow nanoprecipitation process here described. Batch-assisted nanoprecipitation was also performed to evaluate the highlighted benefits of microfluidics. Finally, Z-potential, Dynamic Light scattering, scanning electron microscopy (SEM), and transmission electron microscopy (TEM) were considered to systematically characterize the resulting polymeric nanoparticles. Results demonstrated that monodispersed Eudragit^®^ RS100 NPs under 100 nm are feasible to be produced in a controlled and reproducible approach based on microfluidics. Our investigations confirmed the influence of such critical parameters as the total flow rate and flow rate ratios of the organic and aqueous phases and the concentration of precursor materials on the size and distribution of Eudragit^®^ RS100 nanoparticles. When the process was compared with conventional batch-synthesis of nanoparticles, it was observed that the continuous synthesis using a microreactor was better able to control the particle properties as well as the drug loading and encapsulation efficiency.

## 2. Materials and Methods

### 2.1. Materials

Eudragit^®^ RS100 was generously donated by Evonik Industries AG (Essen, Germany). Absolute ethanol 99.8% (v/v) (EtOH) was purchased from PanReac (Barcelona, Spain). Acetone ≥99.5% and Pluronic F68^®^ were purchased from Sigma-Aldrich (Darmstadt, Germany). All the reagents were used as received. Milli-Q water (conductivity 0.056 µS/cm at 25 °C) used was produced by inverse osmosis using Millipore Elix and filtered through a 0.2-μm membrane. Rifampicin (European Pharmacopoeia (EP) Reference Standard) was purchased from Sigma-Aldrich (Darmstadt, Germany).

### 2.2. Viscosity Measurements

Eudragit^®^ RS100 solutions were prepared at different concentrations of polymer (5% (w/v) and 10% (w/v)) and using different class 3 solvents: ethanol, acetone, and a mixture of EtOH:acetone (1:1). Viscosity of polymeric solutions (η, mPa) were determined at 20 °C using a rotational viscometer Visco Basic Plus (Fungilab, S.A, New York, NY, USA) with a low-viscosity adaptor (LCP). The mean viscosity value and standard deviation of each solution were obtained from the average of four independent measurements.

### 2.3. Synthesis of Eudragit^®^ RS100 Nanoparticles by Conventional Batch Method

**Figure 1:**
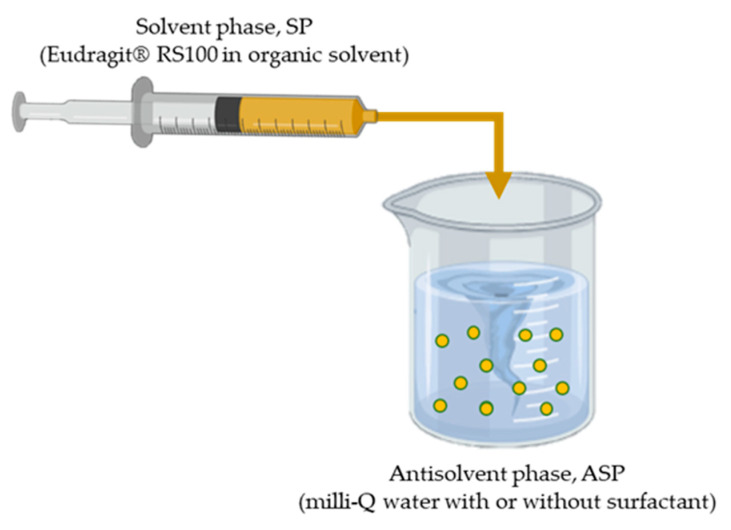
Schematic representation of the conventional batch nanoprecipitation method.

Eudragit^®^ RS100 NPs were obtained by nanoprecipitation using a controlled dropwise addition of the solvent phase (ethanol, acetone and ethanol/acetone) into the antisolvent (Figure 1).

A certain concentration of Eudragit^®^ RS100 (5% and 10% (w/v)) was dissolved in ethanol, acetone and in a mixture of EtOH:acetone (1:1). Then, the polymer solution (solvent phase, SP) was added dropwise (0.5 mL/min) on the corresponding amount of Milli-Q water (antisolvent phase, ASP). The effect of adding the stabilizer Pluronic^®^ F68 was studied, performing the nanoprecipitation process with and without Pluronic^®^ F68 0.5% (w/v), under continuous moderated stirring. The organic solvent was evaporated under magnetic stirring, 600 rpm, overnight at room temperature (23 ± 2 °C). Particles were centrifuged 5 min at 3000 rpm to remove large aggregates. The pellet dispersed in Milli-Q water was collected for further characterization. In this work, different synthesis parameters were studied (polymer concentration, solvent, solvent ratio, surfactant effect), as shown in Table 1.

### 2.4. Synthesis of Eudragit^®^ RS100 Nanoparticles by Micromixer-Assisted Nanoprecipitation

**Figure 2:**
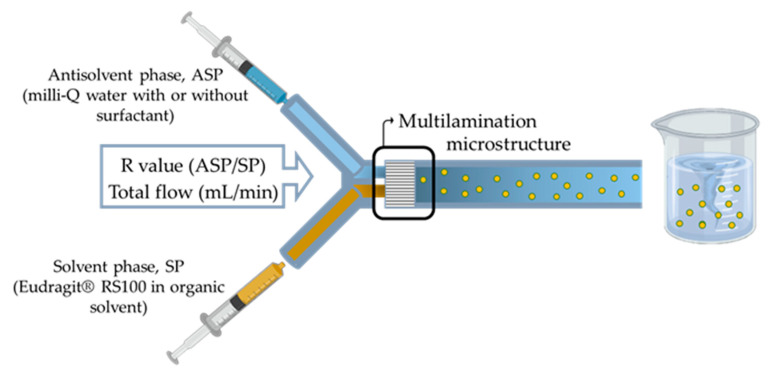
Schematic representation of the micromixer-assisted nanoprecipitation method and the main variables attuned to control the particle size distribution.

Eudragit^®^ RS100 NPs were synthesized by nanoprecipitation in a micromixer device by introducing two streams, SP and ASP (Figure 2) into the mixer.

**Figure 3:**
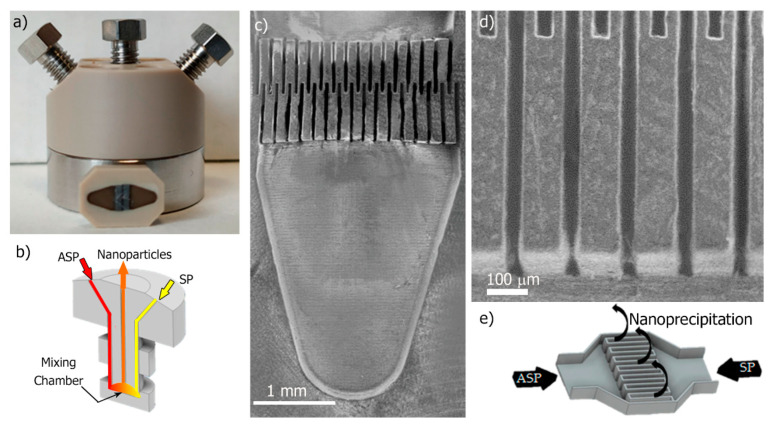
(**a**) Optical image of the interdigital multilamination micromixer, housing and mixing unit; (**b**) Scheme of the ASP and SP inlet stream distribution and the resulting nanoprecipitated stream; (**c**,**d**) Scanning Electron Microscopy images of the inner microstructure of the micromixer unit; (**e**) Scheme to show that the multilamination promotes mixing by diffusion-driven mass transfer.

A polyether ether ketone (PEEK) interdigital multilamination micromixer (Micro4 Industries GmbH, Mainz, Germany) was selected to promote an efficient mixing within the millisecond timeline [46]. The micromixer is depicted in Figure 3a, each inlet (ASP and SP) flow diverged into 16 microchannels (Figure 3b) with 45 µm width that enabled a fast multilamination (Figure 3c,d), and finally, laminated streams flowed out following a 90° trajectory (Figure 3e). Different polymer concentration solutions (Table 2) were prepared by dissolving Eudragit^®^ RS100 in ethanol, acetone, and in a mixture of EtOH:acetone (1:1) (solvent phase, SP). The solution was filtered through a 0.45-µm filter (Millipore^®^ PTFE syringe filter, Thermo Fisher Scientific, Waltham, MA, USA) to prevent clogging. The aqueous phase consisted of Milli-Q water (antisolvent phase, ASP). Both ASP and SP were filled in a syringe and injected in the micromixer using 1/16-inch OD PTFE tubing and a syringe pump (Harvard Apparatus, Holliston, MA, USA). Mixing between both phases was carried out at different ASP/SP flow ratios (R = ASP flow rate/SP flow rate) and total flow ratios.

The resulting nanoprecipitated sample was collected at the outlet of the microfluidic device and the organic solvent was evaporated under magnetic stirring, 600 rpm and overnight at room temperature. Nanoprecipitation experiments were conducted using the micromixer, varying the polymer concentration, the type of solvent, total flow rate, and R ratio as it is given in Table 2.

### 2.5. Rifampicin Encapsulation in Eudragit^®^ RS100 Nanoparticles

Rifampicin-loaded Eudragit^®^ RS100 NPs were prepared by both the conventional batch method and the micromixer-assisted nanoprecipitation method. Rifampicin was added to the solvent phase, that is, polymer and drug were dissolved in EtOH:acetone (1:1). By the batch methodology, a concentration of 5% (w/v) of Eudragit RS100 and rifampicin (50%, 20%, 10%, and 5% (w/w) referred to the polymer weight) were dissolved into the solvent EtOH:acetone. One mL of this organic phase was added dropwise (0.5 mL/min) onto 5 mL of Milli-Q water. On the other hand, the rifampicin loading by the micromixer-assisted nanoprecipitation method was performed by preparing an SP with a concentration of 5% (w/v) of Eudragit^®^ RS100 and rifampicin in EtOH:acetone at the optimized concentration used in the batch-based approach. The ASP consisted of Milli-Q water and both SP and ASP solutions were filled into syringes to be injected by the procedure previously described in Section 2.4. The injection of SP and ASP was carried out at an ASP/SP ratio R of 5 and a total flow rate of 5 mL/min. In both approaches, rifampicin-loaded Eudragit^®^ RS100 NPs were collected and the organic solvent evaporated under magnetic stirring, 600 rpm overnight at room temperature.

### 2.6. Nanoparticle Characterization

#### 2.6.1. Determination of the Entrapped Rifampicin in Eudragit^®^ RS100 Nanoparticles

To determine the amount of encapsulated rifampicin in the Eudragit^®^ RS100 NPs, NPs were washed several times using an Amicon^®^ Ultra-15 centrifugal filter unit with 30 kDa of cutoff. The supernatant was analyzed by UV–Vis spectrophotometry (Jasco V670, Madrid, Spain) at a wavelength of 334 nm to indirectly determine the amount of unencapsulated rifampicin remaining in the nanosuspension by mass balance. Previously, a calibration curve was performed from 0 to 25 ppm of rifampicin. The measurements were performed in triplicate. Encapsulation efficiency (EE) and drug loading (DL) were calculated using mass balances according to (1) and (2), respectively.

(1) $$\mathrm{EE}\;\left(\%\right)=\frac{\text{Initial Rif added}-\text{measured Rif}}{\text{Initial Rif added}}\times 100$$

(2) $$\mathrm{DL}\;\left(\%\right)=\frac{\text{Initial Rif}-\text{measured Rif}}{\text{mg of particles}}\times 100$$

#### 2.6.2. In vitro Release Study of Rifampicin from Nanoparticles

The in vitro release study of rifampicin was performed by introducing Eudragit^®^ RS100 nanoparticles prepared by the conventional batch method and the nanoparticles prepared by the micromixer-assisted nanoprecipitation method at a concentration of 1 mg/mL in the release medium (PBS 1x). Nanoparticles were incubated in an orbital shaker at 37 °C. At specific time intervals, the supernatant was collected using an Amicon^®^ Utra-15 centrifugal filter unit with 30 kDa of cutoff and replaced with fresh PBS to maintain sink conditions. The concentrations of rifampicin in the samples were determined by UV–Vis spectrophotometry at 334 nm. Three different experiments were performed for each formulation.

#### 2.6.3. Size Distribution and Zeta-Potential Measurements

Nanoparticle size, index of polydispersity, and surface charge were studied. All these parameters were measured by Dynamic Light Scattering (DLS) (Zeta Plus; Brookhaven Instruments Corporation, Holtsville, NY, USA). Eudragit^®^ RS100 NP colloid was diluted with Milli-Q water and their particle size distribution and zeta potential were determined at a constant temperature of 25 °C at pH 6 (using a KCl buffer to provide constant ionic strength). At least three measurements of each sample were carried out. Index of polydispersity was calculated by the following Expression (3):

(3) $$\mathrm{PDI}={\left(\frac{\sigma }{\bar{x}}\right)}^{2}$$

where PDI indicates the dispersion of the sample, considering a value lower than 0.1 the limit to consider a monodisperse sample. $\bar{x}$ stands for the mean value of the size and σ^2^ is the variance.

#### 2.6.4. Transmission Electron Microscopy (TEM) Characterization

Morphology and shape characterization were carried out using a T20-FEI transmission electron microscope (FEI Company, Hillsboro, OR, USA). TEM samples were prepared by depositing 40 μL of NP colloid dispersed in Milli-Q water onto a formvar-coated copper TEM grid and then dried for at least 2 h. Polymer samples were negatively stained with phosphotungstic acid following a previous protocol of our group [50]. The nanoparticle size distribution histogram was calculated measuring the NP diameter of TEM images with ImageJ software and using statistical analysis (N = 150).

#### 2.6.5. Scanning Electron Microscopy Characterization

Eudragit^®^ RS100 NPs were visualized using an Inspect F50 field emission gun scanning electron microscope (Eindhoven, the Netherlands) at 10 kV. To visualize them, samples were previously sputtered with a nanocoating of Au/Pd (Leica EM ACE200, Wetzlar, Germany). The nanoparticle size distribution histogram was obtained by following the same protocol as the one aforementioned in the TEM characterization.

#### 2.6.6. Statistical Analysis

Ordinary one-way and two-way analysis of variance (ANOVA) was used to analyze significant size and zeta-potential differences among parameters under study using Prism 7 software (GraphPad Software, San Diego, CA, USA). Statistically significant differences were considered when *p* ≤ 0.05.

## 3. Results and Discussion

Considering that the main aim of this work was to identify the key parameters governing the nanoprecipitation of Eudragit^®^ RS100 polymer and to propose a continuous flow approach to circumvent current limitations of batch production, this section is divided into four distinct parts: (1) Selection of class 3 solvents, (2) Analysis of the results obtained by the conventional batch method, (3) Analysis of the results obtained by the micromixer-assisted nanoprecipitation and synthesis optimization, and (4) Study of rifampicin loading and entrapment efficiency by batch and micromixing-assisted approaches.

### 3.1. Selection of Class 3 Solvents

The solubility of the polymer in the SP and the solution viscosity are key parameters to favor a controlled production of NPs by the nanoprecipitation approach. Therefore, the polymer solubility was studied, the proportion of solvents in the SP, as well as the inherent viscosity in the final dispersive medium. As aforementioned, polymer solvents should fulfill several requirements: be miscible with water, have a low boiling point, and possess minimal toxicity (class 3) [38]. In this study, the tentative solvents that satisfied previous premises were ethanol, acetone, and a mixture of both of them. When the Eudragit^®^ RS100 polymer was dissolved in ethanol, a heterogeneous suspension was obtained in which the polymer was not feasible to be dissolved; for this reason, ethanol was discarded as solvent. On the other hand, acetone and a solvent mixture EtOH:acetone in the volumetric ratio 1:1 were found to successfully dissolve the Eudragit^®^ RS100 polymer. Table 3 summarizes the viscosity of pure solvents and different SPs considered in this work.

### 3.2. Analysis of the Results Obtained by the Conventional Batch Method

Experiments were first conducted in a batch mode, which is the conventional and widely used method in the literature to produce polymeric NPs by the nanoprecipitation approach. Furthermore, in the subsequent sections, these results will be benchmarked with the results achieved in a continuous synthesis mode.

The influence of the polymer concentration in the synthesis was evaluated by the characterization of two different concentrations, 5% and 10% (w/v) of Eudragit^®^ RS100. As can be seen in Table 4, there was a slight influence of the polymer concentration on the resulting NP size, but it was significant (*p* < 0.05). The size of the NPs varied from 62.2 ± 25.6 nm to 53.8 ± 17.9 nm when the concentration was reduced from 10% to 5% (w/v), respectively. These results are in agreement with previous studies related to polymeric NP production by nanoprecipitation where it is accepted that a SP with a high polymer concentration yields larger NPs because: (1) the increase of viscosity reduces the velocity of diffusion of the organic solvent through the antisolvent. This effect is of paramount importance, since the nanoprecipitation method relies on the rapid diffusion of the solvent into the antisolvent, which thereby provokes polymer supersaturation and aggregation in the form of colloidal NPs [52], and (2) the number of polymer molecules per unit volume of solvent favors the nuclei growth as the polymer concentration increases [53]. On the other hand, the use of different solvents yields different NP sizes. It has been reported that the solvent–antisolvent interaction influences the final particle size, probably being governed by the ratio of diffusion of the solvent phase into the antisolvent phase. However, in this particular case, the diffusion into the interface of the organic solvent was not decisive for the final size because no significant differences were found between both diffusion coefficients (D_acetone_ 1.28 × 10^−5^, D_EtOH_ 1.24 × 10^−5^ cm^2^·s^−1^ at finite dilution in water at 20 °C) [51]. On the other hand, the viscosity of the organic solvent is also considered another critical parameter for controlling nanoparticle size [52]. According to the Eudragit^®^ RS100 nanoprecipitation results with different solvents (Table 4), there was a remarkable decrease (*p* < 0.05) of the particle size from 62.2 ± 25.6 nm to 27.9 ± 11.4 nm when the solvent was changed from the mixture of EtOH:acetone to acetone. These results evidence that viscosity is a critical parameter, since an approximate 2-fold increase in SP viscosity (η_acetone_ 3.61, η_EtOH:acetone_ 6.08, mPa·s at 20 °C for Eudragit^®^ RS100 10% (w/v), Table 3) can reduce by half the nanoparticle size in this particular case. Thus, from these results it can be inferred that low SP viscosity promotes a fast mixing and yields small nanoparticles, although the mixing efficiency in the batch-assisted process seemed not be improved and a similar PDI (0.17) was observed (Table 4).

Although it has been reported that the use of surfactants in the synthesis of polymeric NPs by nanoprecipitation methodology is not necessary [54], we also analyzed in this study the presence of an amphiphilic non-ionic surfactant because it can reduce the surface tension between the SP and ASP and accelerate mixing [55]. Thus, production of polymeric NPs was faced using either pure Milli-Q water or Pluronic 0.5% (w/v) in water as the ASP. The surfactant concentration was selected based on previous results [55], where it attained a compromise to reduce the surface tension during the mixing process, but without promoting the surfactant self-assembly above the critical micelle concentration (∼1% w/v) [56]. Working above the critical micelle concentration can result in a heterogeneous particle size distribution constituted not only of Eudragit^®^ RS100 NPs but also of Pluronic-based micelles. While studying the influence of the surfactant on nanoparticle formation, it was observed that the addition of the surfactant had no significant effect (*p* > 0.05) on particle size. This result was observed for both polymer concentrations tested, and confirmed that surfactant is not required, and that interfacial tension does not significantly influence the nanoprecipitation process [57].

**Figure 4:**
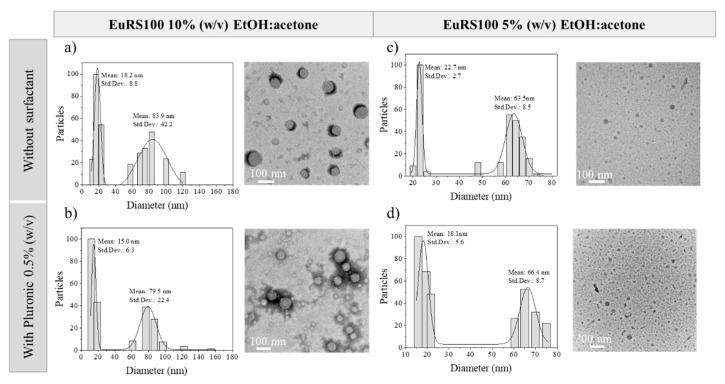
DLS size distribution, representative TEM (**a**,**b**) or SEM (**c**,**d**) images of the resulting nanoparticles. (**a**) Eudragit^®^ RS100 10% (w/v) EtOH:acetone, R = 5, without surfactant, (**b**) Eudragit^®^ RS100 10% (w/v) EtOH:acetone, R = 5, Pluronic 0.5% (w/v), (**c**) Eudragit^®^ RS100 5% (w/v) EtOH:acetone, R = 5, without surfactant, and (**d**) Eudragit^®^ RS100 5% (w/v) EtOH:acetone, R = 5, Pluronic 0.5% (w/v).

According to the literature [58], the degree of mixing and concentration gradients in the batch-assisted nanoprecipitation approach are highly heterogeneous. As a result, although a homogenous nucleation event occurs, the subsequent growth of the nuclei is not homogenous because the polymer concentration profile is not homogeneous in the solution. After the analysis of the particle size distributions of each synthesis condition considered in this work, it was observed as a general trend that there were present two perfectly differentiated NP populations (Figure 4). This evidence was more acute in the case of adding the solvent phase (SP) with a high polymer concentration because of its high viscosity. Thus, in the Eudragit^®^ RS100 10% (w/v) EtOH:acetone conditions (Figure 4a), one population having a mean particle size centered at 83.9 ± 42.2 nm and another one at 18.2 ± 8.8 nm were observed. On the other hand, two separated populations sized 63.5 ± 8.5 nm and 22.7 ± 2.7 nm were also observed in the case of using Eudragit^®^ RS100 5% (w/v) EtOH:acetone conditions (Figure 4c). The results suggested a common particle diameter size of around 20 nm for both conditions, these particles probably having grown in the most efficient mixing locations in the batch reactor and demonstrated the inherent unavoidable concentration gradients in the batch reactor even under stirring. In addition, a second population sensitive to the polymer concentration was identified, which could be produced at different nucleation/growth events as a result of inefficient mixing and heterogeneous polymer concentration profiles. Finally, Figure 4 also reveals that the presence of surfactant did not affect the presence of a multimodal population, as previously described.

### 3.3. Eudragit^®^ RS100 NP Synthesis Based on the Micromixer-Assisted Nanoprecipitation: Study of Parameter Effects

The interdigital multilamination micromixer selected in this work was well-considered to produce polymeric NPs by nanoprecipitation [43] and emulsification [46,50] approaches because of its efficient mixing and high NP throughput (Figure 3). ASP and SP were introduced by two different inlets (Figure 3a,b) and then fluid lamellae of 45 μm were alternatively staggered by 16 microchannels (Figure 3c,d). The mixing of SP into ASP was driven by diffusion mass transfer, being enhanced by a high contact surface between the lamellae and the subsequent flow focusing section where a high shear stress was achieved [46]. The fluid dynamics of the micromixer was varied by using two different total flow rates, a flow rate of 5 mL/min and a 10–fold higher flow rate of 50 mL/min. Considering that the micromixer inner volume was 8 μL, the residence time can be considered to vary between 96 ms and 9.6 ms, at 5 mL/min and 50 mL/min, respectively. The SP stream was composed of Eudragit^®^ RS100 at a concentration of 5% (w/v) or 10% (w/v) in acetone or EtOH:acetone. On the other hand, the ASP stream was just Milli-Q H_2_O and the use of surfactants was discarded because of the lack of influence observed in the nanoprecipitation when using the conventional batch method. Finally, the ratio between ASP/SP (R) streams was also varied to study the variation of NP size with the supersaturation.

#### 3.3.1. Influence of Polymer Concentration

**Figure 5:**
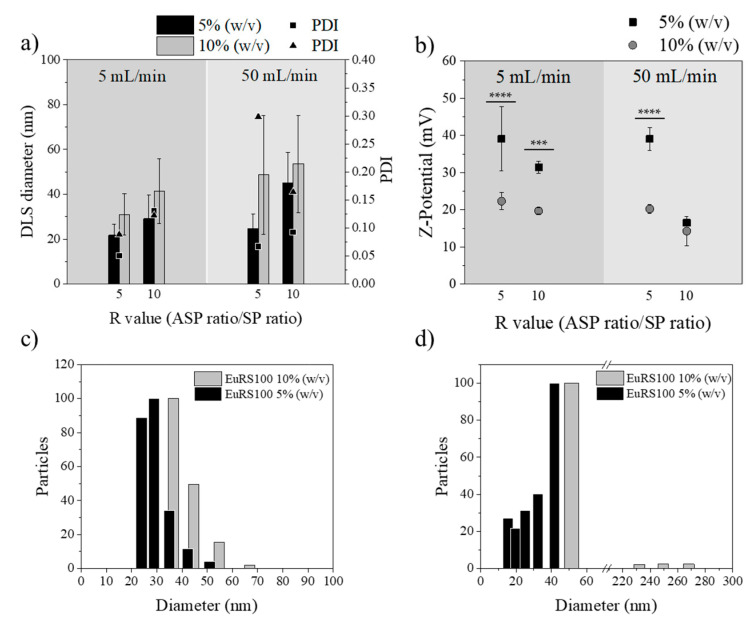
(**a**) DLS diameter of Eudragit^®^ RS100 5% (w/v) EtOH:acetone (black) and Eudragit^®^ RS100 10% (w/v) (gray) of different R values and total flows synthesis (n = 3). Polydispersity index (PDI) of Eudragit^®^ RS100 5% (w/v) EtOH:acetone (■) and Eudragit^®^ RS100 10% (w/v) (▲). (**b**) Z-potential of Eudragit^®^ RS100 5% (w/v) EtOH:acetone (black ■) and Eudragit^®^ RS100 10% (w/v) (gray ●) (*** *p* < 0.001, **** *p* < 0.0001). (**c**) DLS size distribution of synthesis with R value = 10, total flow of 5 mL/min; (**d**) DLS size distribution of synthesis with R value = 10, total flow of 50 mL/min.

Figure 5 shows the effect of the polymer concentration on the final NP size. The polymer concentrations tested were 5% and 10% (w/v), varying also the total flow rate (5 and 50 mL/min) and R values (5 and 10). Similar to the results detailed in the nanoprecipitation by the conventional batch method, the polymer concentration had also a strong influence on the micromixer-assisted nanoprecipitation. As expected, an increase in the polymer concentration produced an increase in nanoparticle sizes (Figure 5a). These results were consistent with previously reported results [59,60] and could be rationalized by following the same arguments as the ones used for nanoprecipitation using the conventional batch method. Based on the mechanism of nucleation and growth, growing events are promoted when there are more polymer chains in the solvent. The polymer concentration determines the number of nuclei, and when the polymer concentration exceeds the supersaturation level, there are more polymeric molecules dissolved in the solvent phase available for NP growth. In addition, the flocculation phenomenon is more likely to occur when the polymer concentration is high, promoting the formation of large particle agglomerates. Furthermore, considering the results shown in Table 3, viscosity had an important effect on the relation between polymer concentration and nanoparticle size. When the polymer concentration increased from 5% (w/v) to 10% (w/v), the viscosity increased. The viscosity increase can reduce the velocity of diffusion of the organic solvent through the antisolvent, and then originates concentration gradients that result in heterogeneous nanoparticle size distributions.

The standard deviation of the NP size was larger when the polymer concentration was 10% (w/v) than when it was 5% (w/v). This result confirms that molecular mixing is difficult to achieve even with this type of efficient micromixer. It was also remarkable that, independent of the polymer concentration, the NP size heterogeneity was even larger at the highest flow rates (Figure 5c,d). This was contradictory to the micromixer fluid dynamics [46], where a high degree of mixing and then uniformity in NP sizes can be achieved at high flow rates. These unexpected results can be explained by a mismatch between the nucleation and growth events. In particular, the short residence time (9.6 ms) achieved with the total flow rate of 50 mL/min promoted a fast nucleation process, but the growth event could not be fully completed at the micromixing unit, and NPs could have evolved in the collection vial. Consequently, polydispersity index values were lower as the polymer concentration and flow rate decreased.

Figure 5b depicts the Z-potential analysis at the two different polymer concentrations. The same behavior was observed in all cases, positive surface charge increased as the NP diameter decreased. Thus, it also probably offered extra stability to these small NPs that prevent the growth of the particles through charge repulsion effects, preventing the aggregation of the formed nuclei and, consequently, the formation of larger particles. The surface charge of the particles can be influenced by several properties of the dispersion media (i.e., pH, solvent composition, ionic strength) and by the presence of adsorbed molecules. However, in this particular case, those variables were not varied and therefore surface charge should not be modified. According to the statistical analysis performed, the surface charge or Z-potential was significantly different (*p* < 0.05) when the size of Eudragit^®^ RS100 NPs was varied. This effect can be explained by the perturbation of the diffuse charge density from particle motion, which in turn is sensitive to the particle size [61,62]. In this sense, Brownian motion increases with decreasing particle size, and the perturbation should be significant. On the other hand, it is well accepted that a higher absolute value of zeta potential means a higher stable state of colloidal systems, and zeta potential values higher than +30 mV enable a stable suspension. Nanoparticles with a zeta potential lower than +30 mV tend to agglomerate easier than those with a zeta potential value above this threshold. Thus, it seems reasonable to get size-dependent surface charging. Other authors have also evidenced this effect. For instance, Holmberg et al. [63] observed that, as the TiO_2_ nanoparticle size decreases, the surface charge density increases, particularly for particles of a diameter less than 10 nm. Finally, it should be pointed out, since it is in agreement with our results, that the chemical modification of polymers with positively and negatively charged groups is reported as a method of controlling the nanoparticle size, preventing their growth by the nanoprecipitation technique [64]. Additionally, it is worth noticing that both the mucoadhesive and cellular uptake properties of Eudragit^®^ RS100 NPs can be attuned by controlling the NP size.

Table 5 summarizes the effects of varying the polymer concentration on the particle size distribution of both nanoprecipitation technologies studied in this work, batch and micromixing. Clearly, micromixing improved the mixing in the nanoprecipitation environment in comparison with the batch procedure, and small NPs with a small PDI were achieved. At a Eudragit^®^ RS100 polymer concentration of 5% (w/v) the mean size and PDI decreased 48.7% and 47.1%, from batch to micromixing nanoprecipitation, respectively.

#### 3.3.2. Influence of the Type of Solvent

**Figure 6:**
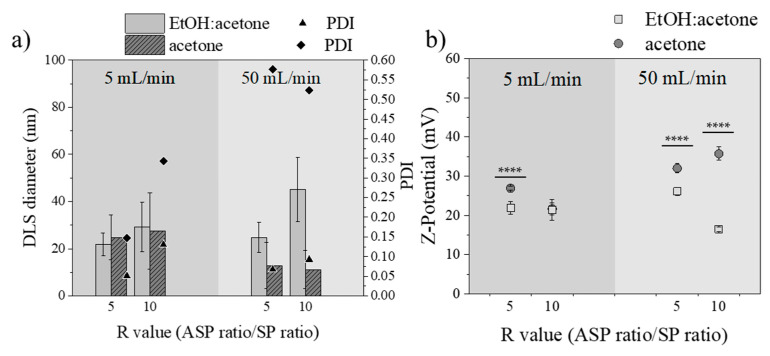
(**a**) DLS diameter of Eudragit^®^ RS100 5% (w/v) EtOH:acetone (light gray) and Eudragit^®^ RS100 5% (w/v) acetone (dark gray) of different R values and total flows synthesis (n = 3). Polydispersity index (PDI) of Eudragit^®^ RS100 5% (w/v) EtOH:acetone (▲) and Eudragit^®^ RS100 5% (w/v) acetone (♦), (**b**) Z-potential of Eudragit^®^ RS100 5% (w/v) EtOH:acetone (light gray ■) and Eudragit^®^ RS100 5% (w/v) acetone (dark gray ●) (**** *p* < 0.0001).

Ethanol and acetone were selected as potential solvents because of the requirements of the nanoprecipitation method: green solvents with a low toxicity and boiling temperature, which also must dissolve Eudragit^®^ RS100 as well as be miscible with water. In agreement with the nanoprecipitation by the conventional batch method and with the literature [65], micromixing-assisted nanoprecipitation results showed the same solvent effect. When the solvent used was acetone, nanoprecipitation yielded small-sized NPs (Figure 6a). Also, the polydispersity index was the lowest when the solvent used was EtOH:acetone, as can be concluded from Figure 6a. Similarly, the density of surface charge of the nanoparticles (Figure 6b) produced with acetone was higher than that produced with the solvent mixture EtOH:acetone, reaching values of +25 mV.

Table 6 summarizes the effects of varying the type of solvent used as ASP on the particle size distribution of both nanoprecipitation technologies studied in this work, batch and micromixing. The most efficient mixing achieved either in acetone or EtOH:acetone by the use of a micromixer resulted in a more homogenous nucleation/growth environment with smaller NPs and PDIs achieved than when using a batch-type procedure.

#### 3.3.3. Influence of Total Flow Rate

**Figure 7:**
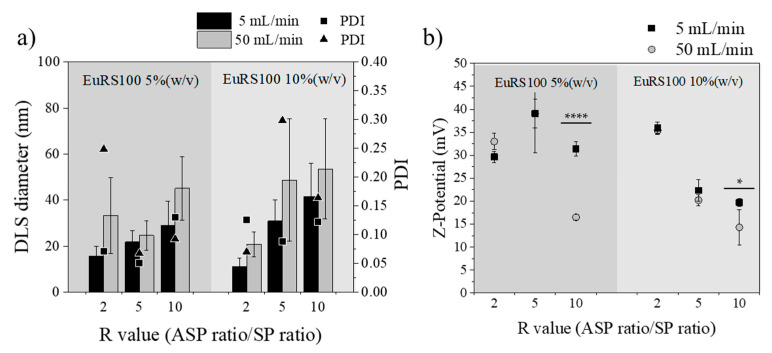
(**a**) DLS diameter of Eudragit^®^ RS100 5% (w/v) and 10% (w/v) EtOH:acetone of total flow 5 mL/min (black) and 50 mL/min (gray) of different R values (n = 3). Polydispersity index (PDI) of 5 mL/min (■) and 50 mL/min (▲), (**b**) Z-potential of 5 mL/min (black ■) and 50 mL/min (gray ●) (**** *p* < 0.0001), (* *p* < 0.05).

**Figure 8:**
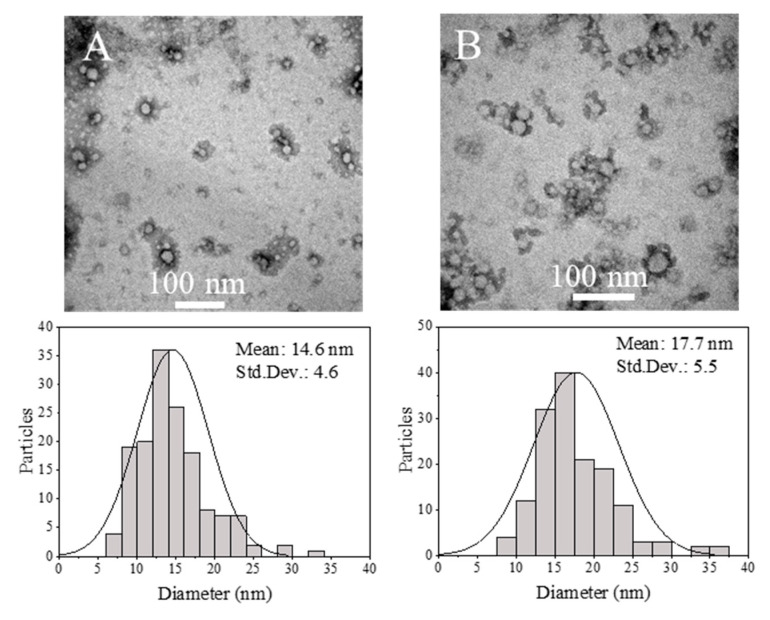
TEM micrographs and size distribution histograms of the synthesis polymer concentration of Eudragit^®^ RS100 5% (w/v), R (ASP/SP) value = 10: (**a**), 5 mL/min, (**b**) 50 mL/min.

The influence of the total flow rate was investigated by comparing the following variables: (1) polymer concentration (5% and 10% (w/v)) dissolved in EtOH:acetone, and (2) R, solvent/antisolvent ratios (2, 5 and 10). Figure 7 depicts the effects of the total flow rates of ASP and SP in the micromixer, and then the residence time, on the particle size distribution of resulting Eudragit^®^ RS100 NPs. In this section only the experiments where the SP was a mixture of EtOH and acetone were considered, but as it was observed in Figure 4, Figure 5 and Figure 6, the tendency was similar when acetone was the unique solvent in the SP stream. Unexpectedly, the smallest and most homogenous Eudragit^®^ RS100 NPs were obtained at a total flow rate of 5 mL/min instead of 50 mL/min. This observation was ubiquitous for any R and polymer concentration here considered (*p* ≤ 0.05). Hydrodynamic modelling of this type of micromixer has resulted in the improvement of the mixing efficiency as the total flow rate was increased [43,46], which is not in agreement with the results here obtained. This surprising result was reproduced several times, certifying that it was inherent to the nanoprecipitation synthesis approach. The polydispersity index was also higher at the flow rate of 50 mL/min than that of 5 mL/min. TEM micrographs and size analysis in Figure 8 also corroborated the previous findings by DLS. It can be inferred from these achievements that the NP size heterogeneity is highly dependent on the residence time at the micromixing unit. Since polymer precipitation obeys the classical nucleation–growth theory, the final particle size is governed not only by the nucleation event but also by the growing process and the aggregation phenomena. While it is conventionally considered that small mean nanoparticle sizes and homogenous size distributions require a fast nucleation event and efficient mixing, a controllable growth should not be ignored. In this particular case, the residence time at the total flow rate of 50 mL/min was estimated to be 9.6 ms (10 times more than at 5 mL/min). Thus the need of obtaining an efficient mixing and controlled nucleation by increasing the total flow rate could misguide our aim to control the final NP size. In all the cases, the outlet stream of the micromixer was collected in a vial where the mixing was not as efficient as in the micromixer. The mismatch between nucleation and growth events can promote the uncontrollable growth of nuclei and even a secondary nucleation [66] in the collection vial. As a result, the nucleation event at 9.6 ms could be better controlled than that at 96 ms, but the growth event was badly controlled in a vial, while in the micromixer the concentration profiles were minimized, yielding NPs with a small PDI and narrow size distribution. It is of paramount importance to notice that mixing is required for both nucleation and growth, and that residence time should be high enough to lead to complete nucleation/growth events. On the other hand, Z-potential is also sensitive to the residence time considered. Generally, the most positively charged NPs were obtained in the synthesis conditions where NPs were the smallest in size. This fact is in agreement with our previous explanation of the size-dependent surface charging.

#### 3.3.4. Influence of R Ratio Value

**Figure 9:**
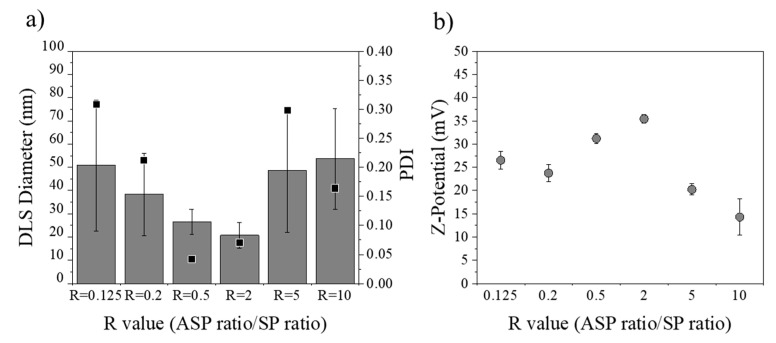
(**a**) DLS diameter and polydispersity index (PDI) of Eudragit^®^ RS100 10% (w/v) EtOH:acetone of different R values at 50 mL/min of total flow (n = 3), (**b**) Z-potential of Eudragit^®^ RS100 10% (w/v) EtOH:acetone of different R values at 50 mL/min of total flow.

The solubility of Eudragit^®^ RS100 in the final nanoprecipitation mixture (supersaturation) was crucial to control the size of NPs. Therefore, the effect of the solvent concentration defined by the R ratio (ASP/SP) was further investigated. Figure 9 summarizes the results obtained after the analysis of the R value and its effect on the NP size and surface charge. To streamline the analysis, representative synthesis conditions were considered: polymer concentration of 10% (w/v) dissolved in EtOH:acetone and a total flow rate of 50 mL/min. The R value, the coefficient between the aqueous phase (ASP) and the organic (SP), was varied from 0.125 to 10. The size of Eudragit^®^ RS100 NPs produced by nanoprecipitation exhibited minimum R values in the range of 0.5–2, with a value of polydispersity under 0.1 (i.e., uniform size distribution) (Figure 9a). On the other hand, when the nanoprecipitation process was performed with a high amount of solvent or antisolvent phase, the PDI and final NP size increased. This was for instance the case of R = 0.125 or R = 10 (Figure 9a), where, although the solvent concentrations were very different, the size remained constant with no significant difference between R = 0.125 and R = 10 (*p* ≤ 0.05). The mechanism that governed this effect was unclear, a plethora of different conclusions and theories existing in the literature [52,59,67]. As aforementioned, two trends can be highlighted, depending on the time-scale particle formation [68]. When the solvent phase fraction is high (R < 0.5), the supersaturation is low and nucleation events are not favored. As a result, the nuclei density is low and the local polymer concentration is high, favoring growth events and large NP sizes. On the other hand, when the antisolvent phase fraction is high (R > 5), the polymer solubility is drastically reduced, and nuclei density is high. However, although the local polymer concentration should be small enough to yield the smallest NPs, the aggregation of NPs is highly favored, resulting in heterogeneous and large NPs. As a result, it is the balance between nucleation rate and aggregation (R = 0.5–2) that further controls the production of monodisperse and small-sized Eudragit^®^ RS100 NPs. On the other hand, as was highlighted in the results of the previous section, the value of the Z-potential of smaller particles is higher than the value of larger ones (Figure 9b). This fact may also influence the colloidal stability and the final nanoparticle size observed at R values of 0.2–5.

### 3.4. Study of Rifampicin Loading and Entrapment Efficiency by Batch and Micromixing-Assisted Approaches

**Figure 10:**
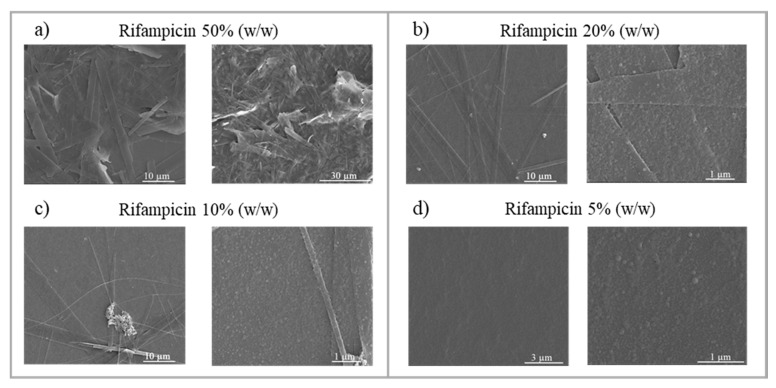
SEM visualization of rifampicin crystal formation at different rifampicin concentrations: (**a**) 50% (w/w), (**b**) 20% (w/w), (**c**) 10% (w/w), and (**d**) 5% (w/w) referred to polymer. The synthesis analyzed was: Eudragit^®^ RS100 5% (w/v) EtOH:acetone by batch methodology (R = 5).

Rifampicin is an example of a drug whose administration is negatively influenced by its hydrophobicity [69]. As such, rifampicin was here considered as an example of the hydrophobic drug concept to study its loading feasibility within the developed NPs. When a new controlled drug delivery system is designed, the main objective is to maximize the amount of encapsulated drug. However, minimizing the drug loss during the synthesis process is required in order to reduce the manufacturing costs. Therefore, the optimal formulation of the study was based on achieving high drug content and drug entrapment while preserving a narrow size distribution. Considering the difficulties that arise when both polymer and drug nanoprecipitation occur, it is required to couple both nanocrystallization processes in order to avoid the formation of segregated free drug crystals that are not entrapped by the polymer. Nanocrystallization kinetics are sensitive to the saturation concentration; thus, a first evaluation of the formation of rifampicin segregated nanocrystals was studied in a conventional batch process to be extended further to the continuous process. In this case, it was not considered appropriate to first use the micromixer-assisted process because uncontrolled production of nanocrystals can irreversibly block micromixer channels having a width of 50 μm. As shown in Figure 10, large rifampicin crystals were precipitated when the rifampicin concentration was higher than 5% (w/w) referred to the polymer weight. The dimension of such crystals was set in the micrometer scale and was comparable to the dimensions of the micromixer channels. Consequently, these results confirmed that nanoprecipitation conditions cannot be directly studied in a microfluidic system. When the concentration of rifampicin was 5% (w/w), no micrometric crystals were observed, suggesting that either rifampicin and polymer nanocrystallization was coupled or that rifampicin was not precipitating because the nucleation was not yet favored. The presence of nanometric rifampicin nanocrystals was not feasible to be conducted, so rifampicin could not be totally loaded inside Eudragit^®^ RS100. At this stage, it should be highlighted that nucleation of polymer and drug can be either homogenous or heterogeneous. The supersaturation required for homogeneous nucleation is much higher than for heterogeneous nucleation. In addition, heterogeneous nucleation can occur because the formed nuclei can reduce the critical free energy for nucleation formulation and it can occur at a lower supersaturation condition [70]. Thus, nanocrystallization of rifampicin can be feasible, even if the homogenous saturation conditions were not achieved.

**Figure 11:**
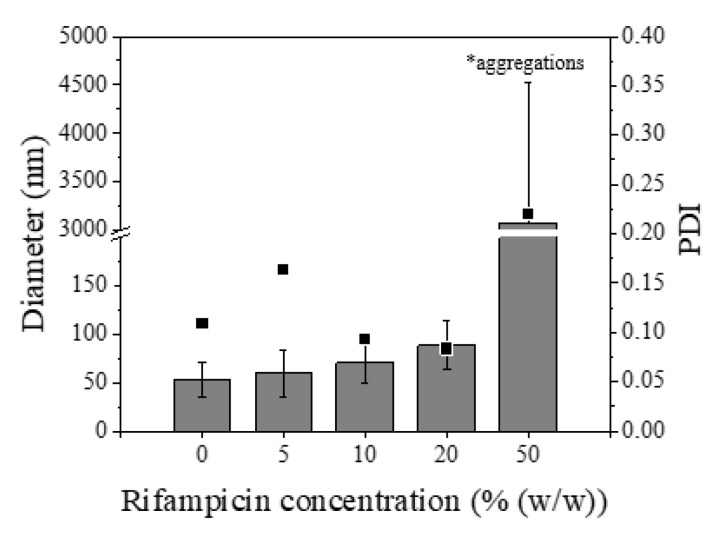
Effect of rifampicin concentration (% (w/w) referred to polymer) on nanoparticle hydrodynamic diameter. Data obtained by DLS measurement. The synthesis analyzed was: Eudragit^®^ RS100 5% (w/v) EtOH:acetone by batch methodology (R = 5).

The average hydrodynamic diameter of the nanoparticles obtained with different concentrations slightly increased from 53.8 nm for empty nanoparticles to 88.8 nm for 20% (w/w) of rifampicin added (Figure 11).

**Figure 12:**
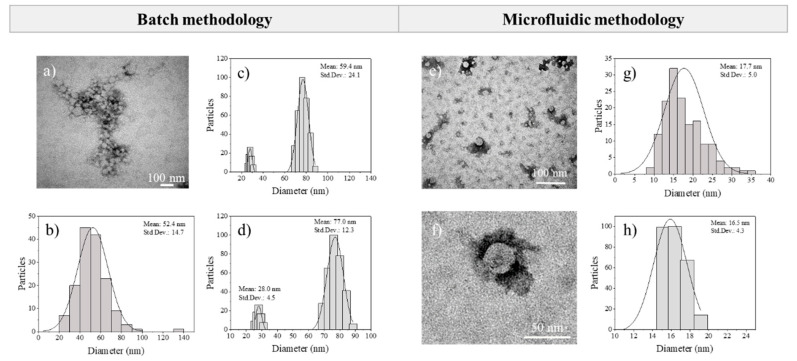
(**a**–**d**) Rifampicin-loaded nanoparticles by batch methodology: (**a**) SEM micrograph, (**b**) size distribution from SEM images, (**c**) DLS size distribution, (**d**) peak analysis of DLS distribution. (**e**–**h**) Rifampicin-loaded nanoparticles by microfluidic methodology: (**e**) SEM micrograph, (**f**) zoomed SEM micrograph, (**g**) size distribution from SEM images, (**h**) DLS size distribution.

Rifampicin encapsulation at 5% (w/w) was set as the proper concentration in order to have better control over the nanoprecipitation process. When the rifampicin was encapsulated by the conventional batch methodology, the mean particle diameter was 52.4 ± 14.7 nm, the drug encapsulation efficiency was 30.1 ± 5.7 wt.%, and the drug loading 1.4 ± 0.3 wt.% (Table 7). The hydrodynamic diameter was similar to one of the unloaded ones (55.3 ± 15.9 nm), but a slight increase in the polydispersity index was observed from 0.11 to 0.17 (Table 7). As depicted in Figure 12c,d, the DLS analysis of Eudragit^®^ RS100 rifampicin-loaded NPs produced by the conventional batch procedure yielded two differentiated size populations, one of 28 ± 4.5 nm and a larger one of 77.0 ± 12.3 nm. This result was in agreement with our previous findings in Section 3.2 and it was rationalized by the existence of different nucleation/growth events because of inefficient mixing and heterogeneous polymer concentration profiles. On the other hand, rifampicin-loaded nanoparticles were also synthesized by the microfluidic methodology (polymer concentration of 5% (w/v) dissolved in EtOH:acetone, a total flow of 5 mL/min and the R value = 5). In this case, as it was noticed for unloaded Eudragit RS100, the mean particle size was smaller and the PDI narrower than the NPs produced by the conventional batch approach (Table 7). The mean particle diameter was 17.7 ± 5.0 nm, observing no significant differences regarding the unloaded nanoparticles produced by the micromixing-assisted process. The same trend was observed with the polydispersity index, a slight increase was found when rifampicin was encapsulated, increasing from 0.05 to 0.07 (Table 7). Regarding rifampicin encapsulation, the encapsulation efficiency was significantly increased from 30.1 ± 5.7% to 42.3 ± 2.4 wt.%, using the conventional and micromixing-assisted nanoprecipitation processes, respectively. The rifampicin loading was also slightly increased when the micromixer was used (2.0 ± 0.14 wt.%). As a result, it seemed that the production of Eudragit^®^ RS100 rifampicin-loaded NPs was favored by the micromixing-assisted nanoprecipitation process.

Figure 12 depicts representative electron microscopy images of Eudragit^®^ RS100 rifampicin-loaded NPs produced by the two approaches considered in this work. Particle size histograms obtained from image treatment and DLS are in agreement and demonstrate the remarkable improvement in the size control when rifampicin-loaded nanoparticles were synthesized by the micromixing-assisted nanoprecipitation.

Nanoparticles produced by the conventional batch nanoprecipitation method and by the micromixer-assisted nanoprecipitation method showed a 19.0 ± 4.7 wt.% and a 15.9 ± 4.6 wt.% rifampicin release in 30 min, respectively. In 48 h almost all the rifampicin content was released (90.9 ± 4.8 wt.% and 90.3 ± 1.8 wt.% for each formulation) (Table 8). The release profiles of both nanoparticle formulations showed an initial burst release followed by a sustained drug diffusion.

Finally, we demonstrated in this study that micromixing-assisted nanoprecipitation is not only an adequate method to control the encapsulation of hydrophobic drugs such as rifampicin, but also a promising method to assure a continuous and scalable production. Batch synthesis protocols have a limited scalability because of the difficulties in assuring good mixing conditions. However, the continuous process here proposed was able to provide a continuous production of rifampicin-loaded NPs with a throughput 1.7 times faster than the conventional batch methodology (2.5 g/h for microfluidic synthesis and 1.5 g/h for batch synthesis). In addition, it improved the encapsulation efficiency and allowed obtaining a narrower size distribution. A high throughput could be easily scaled-out by numbering-up the micromixing units.

## 4. Conclusions

Eudragit^®^ RS100 nanoparticles were produced using the nanoprecipitation-solvent displacement method by a discontinuous and a continuous technique. In this work, an exhaustive study of each influential parameter was developed. Significant differences in NP size and heterogeneity were observed when the polymer concentration, type of solvent, and synthesis technologies were modified. On the other hand, the presence or absence of a surfactant was not observed to be crucial. Two particle populations were observed by DLS analysis in all studies carried out using the conventional batch nanoprecipitation approach. This heterogeneous distribution of NPs was due to the intrinsic uncontrolled mixing of the methodology. As indicated by this study, the production of rifampicin loaded and unloaded Eudragit^®^ RS100 NPs by the micromixing-assisted approach was able to improve the size control and PDI, promoting a higher encapsulation efficiency and drug-loading than the ones obtained in a conventional batch process, as well as a high productivity. This improvement was a result of the excellent mixing achieved in the interdigital micromixer considered in this work. However, the residence time and nucleation/growth events should be balanced in order to harness the microfluidic advantages.

## Figures and Tables

**Table 1:** Parameters and conditions used for the Eudragit^®^ RS100 NP synthesis in batch reactors. R value stands for antisolvent/solvent ratio.

Eudragit^®^ RS100 Concentrations	Solvents	R Value	Surfactant
5% (w/v)	Ethanol	5	Pluronic 0.5% (w/v)
	Acetone		
10% (w/v)	EtOH:acetone (1:1)		No surfactant

**Table 2:** Parameters and conditions used for the Eudragit^®^ RS100 nanoparticle synthesis by microfluidic nanoprecipitation.

Eudragit^®^ RS100 Concentrations	Solvents	Total Flow	R Value
5% (w/v)	Acetone	5 mL/min	2
			5
10% (w/v)	EtOH:acetone (1:1)	50 mL/min	0.5
			0.2
			0.125

**Table 3:** Viscosity values (η, mPa·s) at 20 °C of solvents and polymeric solutions used in this work.

	Eudragit^®^ RS100		
	0% (w/v)	5% (w/v)	10% (w/v)
**Ethanol**	1.07 ^a^	-	-
**Acetone**	0.31 ^a^	1.57 ± 0.04	3.61 ± 0.01
**EtOH:acetone (1:1)**	0.69	4.56 ± 0.16	6.08 ± 0.58

^a^ Values obtained from reference [51].

**Table 4:** Dynamic Light Scattering (DLS) and size mean and Z-potential (at pH 6) characterization of the synthesis obtained by the batch process.

Synthesis	Mean Diameter ± Std.Dev. (nm)	PDI	Z-potential (mV)
EuRS100 10% (w/v) EtOH:acetone	62.2 ± 25.6	0.17	21.3 ± 6.9
EuRS100 10% (w/v) EtOH:acetone, Pluronic 0.5% (w/v)	65.4 ± 25.4	0.15	22.0 ± 7.6
EuRS100 10% (w/v) acetone	27.9 ± 11.4	0.17	19.6 ± 2.5
EuRS100 5% (w/v) EtOH:acetone	53.8 ± 17.9	0.11	13.2 ± 3.5
EuRS100 5% (w/v) EtOH:acetone, Pluronic 0.5% (w/v)	50.6 ± 18.9	0.14	14.32 ± 4.1
EuRS100 5% (w/v) acetone	20.6 ± 9.2	0.20	17.6 ± 0.6

**Table 5:** Mean diameter and PDI comparison between batch and continuous flow production: effect of polymer concentration in the ASP.

Methodology	Synthesis	Mean Diameter ± Std.Dev. (nm)	PDI
By batch (R = 5)	EuRS100 10% (w/v) EtOH:acetone	62.2 ± 25.6	0.17
	EuRS100 5% (w/v) EtOH:acetone	53.8 ± 17.9	0.11
By microfluidic (R = 5, Q = 5 mL/min)	EuRS100 10% (w/v) EtOH:acetone	31.0 ± 9.2	0.09
	EuRS100 5% (w/v) EtOH:acetone	21.8 ± 4.9	0.05

**Table 6:** Mean diameter and PDI comparison between batch and continuous flow production: effect of type of solvent in the ASP.

Methodology	Synthesis	Mean Diameter ± Std. Dev. (nm)	PDI
By batch (R = 5)	EuRS100 5% (w/v) acetone	28.6 ± 12.4	0.19
	EuRS100 5% (w/v) EtOH:acetone	53.8 ± 17.9	0.11
By microfluidic (R = 5, Q = 5 mL/min)	EuRS100 5% (w/v) acetone	24.8 ± 9.5	0.15
	EuRS100 5% (w/v) EtOH:acetone	21.8 ± 4.9	0.05

**Table 7:** Size, DLS mean size, PDI, Z-potential, encapsulation efficiency (EE) and drug loading (DL) of rifampicin-loaded nanoparticles synthesized by batch and by microfluidic methodologies.

Methodology	Drug:polymer	Mean Size (nm)	DLS Mean Size (nm)	PDI	Z-potential (mV)	EE (%)	DL (%)
In batch	-	55.3 ± 15.9	53.8 ± 17.9	0.11	13.2 ± 3.5	-	-
	1:20	52.4 ± 14.7	59.4 ± 24.1	0.17	13.7 ± 7.6	30.1 ± 5.7	1.4 ± 0.3
By microfluidic	-	18.4 ± 5.5	21.8 ± 4.9	0.05	31.9 ± 8.6	-	-
	1:20	17.7 ± 5.0	16.5 ± 4.3	0.07	17.4 ± 2.2	42.3 ± 2.4	2.0 ± 0.1

**Table 8:** Cumulative drug release data of nanoparticles prepared by conventional batch nanoprecipitation method and by micromixer-assisted nanoprecipitation method.

Conventional Batch Synthesis			Total Flow 5 mL/min Synthesis		
Time	Cumulative Amount Released (%)		Time	Cumulative Amount Released (%)	
Hours	Mean	Std. Dev.	Hours	Mean	Std. Dev.
0.5	19.0	4.7	0.5	15.9	4.6
1	36.3	4.4	1	28.4	4.6
2	52.4	5.5	2	37.6	6.5
4	70.2	10.7	4	53.8	8.4
8	87.5	3.9	8	77.4	9.0
24	89.4	3.8	24	89.7	6.6
48	90.9	4.8	48	90.3	1.8
